# Supplementary material for: Characterization of Tunneled Wide Band Gap Mixed Conductors: The Na2O-Ga2O3-TiO2 System
Source: Nanomaterials (Basel). 2023 Jul 12;13(14):2054. doi: 10.3390/nano13142054 (PMC10383386; doi:10.3390/nano13142054)
Supplement: Supplementary file 1 [file nanomaterials-13-02054-s001.zip › nanomaterials-2421761-supplementary.pdf]

nanomaterials

Supplementary Materials

Characterization of Tunneled Wide Band Gap Mixed Conductors: The Na_2O-Ga_2O_3-TiO_2 System

Javier García-Fernández [1], María Hernando [1], Almudena Torres-Pardo [1], María Luisa López [1], María Teresa Fernández-Díaz [2], Qing Zhang [3], Osamu Terasaki [3], Julio Ramírez-Castellanos [1], José M. González-Calbet [1,4]

[1] Inorganic Chemistry Department, Chemical Sciences Faculty, Universidad Complutense de Madrid, 28040 Madrid, Spain
[2] Institut Laue-Langevin, 38042 Grenoble cedex 9, France
[3] Centre for High-Resolution Electron Microscopy, ShanghaiTech University, 201210 Shanghai, China
[4] ICTS National Center for Electron Microscopy, Universidad Complutense de Madrid, 28040 Madrid, Spain

1. Supplementary Materials

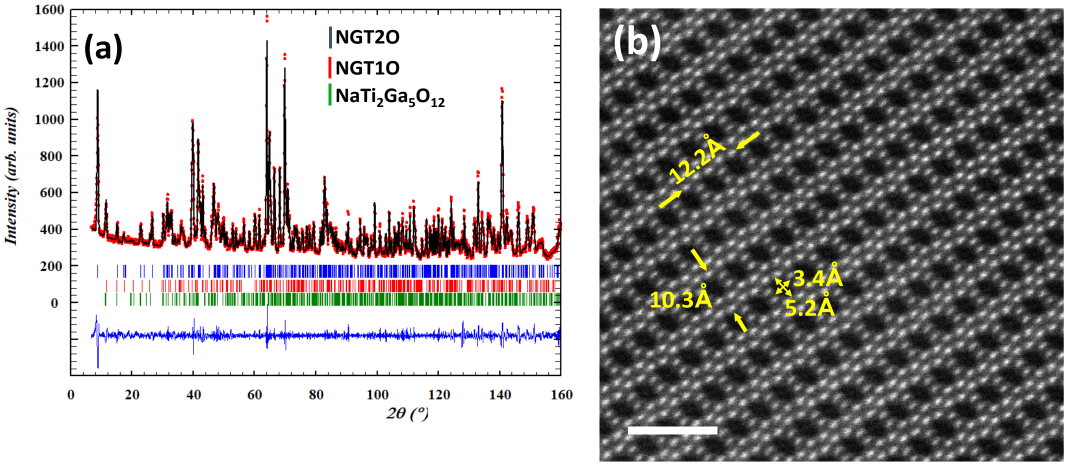

Figure S1. (a) Lebail refinement of the NPD data for NGT2O oxide. The observed patterns (red circles), calculated patterns (continuous black line), and difference curves (continuous blue line) are shown. For the case of NGT2O oxide, NGT1O and $NaTi_2Ga_5O_{12}$ were included as impurities. (b) HAADF-STEM image corresponding to NGT2O sample along [010]. Cell parameters and size of the octagonal tunnel are indicated. The scale bar is 2 nm.

Table S1. Selected interatomic distances (Å) in $Na_{0.80(3)}Ga_{4.66(3)}Ti_{0.34(3)}O_8$ from neutron powder diffraction data.

Atom	Distance (Å)
Ga1-O1x2	1.849(3)
Ga1-O2	1.827(3)
Ga1-O4	1.838(4)
(Ga,Ti)2-O1	1.964(4)
(Ga,Ti)2-O2	2.051(5)
(Ga,Ti)2-O2x2	2.087(2)
(Ga,Ti)2-O3x2	1.9078(18)
(Ga,Ti)3-O3x2	1.9997(20)
(Ga,Ti)3-O4x4	1.9875(20)

Table S2. Selected interatomic distances (Å) in $Na_{0.70(1)}Ga_{4.72(1)}Ti_{2.28(1)}O_{12}$ from neutron powder diffraction data.

Atom	Distance (Å)
Ga1-O1	1. 860(5)
Ga1-O4x2	1.823(3)
Ga1-O5	1.818(3)
Ga1-O4	1.838(4)
(Ga,Ti)2-O1x2	2.037(4)
(Ga,Ti)2-O2	1.982(7)
(Ga,Ti)2-O5x2	1.977(4)
(Ga,Ti)2-O6	1.945(7)
(Ga,Ti)3-O2x2	1.9875(20)
(Ga,Ti)3-O3	1.839(12)
(Ga,Ti)3-O5	2.179(12)
(Ga,Ti)3-O6x2	1.968(8)
(Ga,Ti)4-O3x4	1.976(3)
(Ga,Ti)4-O4x2	1.941(5)

Table S3. Compositional analysis of the NGTOs samples from WDS and experimental formula assuming a complete anionic sublattice.

Atom	Na (% wt.)	Ga (% wt.)	Ti (% wt.)	Experimental Formula
NGT1O	3.4	63.2	3.3	$Na_{0.7}Ga_{4.7}Ti_{0.3}O_8$
NGT2O	3.2	54.3	10.6	$Na_{0.8}Ga_{4.8}Ti_{1.2}O_{10}$
NGT3O	3.1	47.5	16.8	$Na_{0.8}Ga_{4.8}Ti_{2.2}O_{12}$

Table S4. Resistivity, Capacity* and n values obtained from the equivalent circuits fits for selected temperatures for NGT1O.

T (K)	ρ1(MΩ.cm)	C1 (F.cm-1)	n1	ρ2(MΩ.cm)	C2 (F.cm-1)	n2	C3 (F.cm-1)	n3
755	0.55	1.44E-10	0.78	1.01	3.56E-08	0.45	6.34E-07	0.37
750	0.60	1.35E-10	0.79	1.09	3.28E-08	0.46	5.61E-07	0.37
745	0.67	1.28E-10	0.79	1.19	3.05E-08	0.46	5.08E-07	0.37
740	0.68	1.25E-10	0.79	1.18	2.83E-08	0.47	4.53E-07	0.35
735	0.79	1.09E-10	0.80	1.35	2.40E-08	0.47	4.12E-07	0.36
731	0.85	1.03E-10	0.80	1.48	2.20E-08	0.48	3.80E-07	0.36
726	0.95	1.01E-10	0.80	1.61	2.06E-08	0.48	3.46E-07	0.37
720	0.98	9.86E-11	0.80	1.62	2.05E-08	0.48	3.21E-07	0.34
715	1.18	9.74E-11	0.80	1.94	1.91E-08	0.48	3.00E-07	0.36
711	1.22	9.35E-11	0.80	1.97	1.71E-08	0.49	2.70E-07	0.35
706	1.39	7.51E-11	0.82	2.22	1.26E-08	0.51	2.37E-07	0.34
701	1.52	7.83E-11	0.82	2.59	1.28E-08	0.50	2.23E-07	0.36
696	1.77	8.72E-11	0.80	3.00	1.34E-08	0.49	2.09E-07	0.37
691	2.20	8.48E-11	0.80	3.76	1.18E-08	0.49	1.88E-07	0.41
685	2.48	7.52E-11	0.81	4.16	9.63E-09	0.50	1.62E-07	0.41
681	2.80	6.59E-11	0.82	4.50	8.22E-09	0.51	1.45E-07	0.38
676	3.32	6.65E-11	0.81	5.48	7.28E-09	0.52	1.27E-07	0.42

671	3.85	5.93E-11	0.82	6.24	6.44E-09	0.52	1.11E-07	0.42
666	4.64	5.97E-11	0.81	6.84	5.73E-09	0.53	9.46E-08	0.41
661	5.28	5.43E-11	0.82	8.00	5.14E-09	0.53	8.33E-08	0.42
656	6.50	5.96E-11	0.81	9.56	5.57E-09	0.52	7.98E-08	0.43
646	8.25	4.07E-11	0.84	11.94	3.42E-09	0.55	5.46E-08	0.42
641	9.64	4.11E-11	0.83	13.57	3.19E-09	0.56	4.81E-08	0.42
636	11.30	4.38E-11	0.82	14.99	2.97E-09	0.56	4.27E-08	0.43
631	13.16	4.03E-11	0.83	18.78	3.26E-09	0.54	4.09E-08	0.43
626	14.26	3.26E-11	0.85	20.34	2.57E-09	0.56	3.39E-08	0.41
621	18.32	4.25E-11	0.82	22.02	2.82E-09	0.56	3.04E-08	0.42
616	19.11	3.29E-11	0.84	27.91	2.32E-09	0.55	2.74E-08	0.42
611	24.23	3.80E-11	0.82	31.37	2.33E-09	0.56	2.39E-08	0.42
606	25.37	2.89E-11	0.86	33.61	1.76E-09	0.58	1.93E-08	0.39
601	28.10	2.62E-11	0.87	39.25	1.54E-09	0.58	1.72E-08	0.39
596	35.15	2.81E-11	0.85	46.55	1.60E-09	0.57	1.56E-08	0.40
591	43.22	3.15E-11	0.83	59.10	1.70E-09	0.56	1.53E-08	0.43
586	47.79	2.70E-11	0.85	68.59	1.39E-09	0.57	1.33E-08	0.42
581	56.14	2.65E-11	0.85	76.60	1.34E-09	0.57	1.15E-08	0.41
576	69.87	2.87E-11	0.84	89.88	1.36E-09	0.57	1.06E-08	0.42
571	79.99	2.52E-11	0.85	118.19	1.14E-09	0.57	1.01E-08	0.45
565	89.54	2.17E-11	0.87	124.63	9.64E-10	0.59	8.23E-09	0.41
559	118.60	2.68E-11	0.83	196.61	9.68E-10	0.56	1.09E-08	0.52

Table S5. Resistivity, Capacity* and n values obtained from the equivalent circuits fits for selected temperatures for NGT3O.

T (K)	ρ1(MΩ.cm)	C1 (F.cm-1)	n1	ρ2(MΩ.cm)	C2 (F.cm-1)	n2
408	3033.60	1.05E-11	0.82	1996.80	5.63E-10	0.59
413	2983.68	1.02E-11	0.82	1925.76	5.99E-10	0.60
418	2880.00	1.04E-11	0.82	1779.84	6.21E-10	0.59
423	2732.16	1.01E-11	0.83	1741.44	6.41E-10	0.58
428	2576.64	1.06E-11	0.82	1505.28	6.12E-10	0.59
433	2330.88	1.01E-11	0.84	1415.04	5.59E-10	0.59
438	2186.88	1.04E-11	0.83	1297.54	6.96E-10	0.58
443	2000.64	1.06E-11	0.83	1130.69	6.92E-10	0.57
448	1838.59	1.07E-11	0.83	1003.97	8.43E-10	0.57
452	1737.22	1.20E-11	0.81	892.22	1.05E-09	0.57
457	1526.98	1.18E-11	0.82	814.66	1.07E-09	0.55
461	1364.93	1.23E-11	0.82	713.09	1.16E-09	0.55
466	1174.46	1.17E-11	0.84	605.76	1.20E-09	0.56
471	1028.74	1.29E-11	0.82	490.37	1.63E-09	0.54
476	898.18	1.44E-11	0.80	398.02	1.90E-09	0.55
481	735.94	1.37E-11	0.82	343.10	1.87E-09	0.53
486	604.61	1.27E-11	0.84	287.23	2.07E-09	0.52
491	518.59	1.35E-11	0.84	238.46	2.93E-09	0.51
496	442.18	1.63E-11	0.81	191.45	3.78E-09	0.50
500	365.95	1.57E-11	0.82	155.72	3.73E-09	0.50
505	306.82	1.57E-11	0.82	137.79	4.46E-09	0.48
510	252.86	1.40E-11	0.85	116.57	4.14E-09	0.48
515	217.34	1.61E-11	0.83	99.05	5.42E-09	0.47
520	188.46	1.86E-11	0.82	80.51	6.92E-09	0.47
525	163.10	2.08E-11	0.81	67.23	8.59E-09	0.47

530	135.39	1.91E-11	0.82	61.76	9.57E-09	0.45
535	117.03	2.04E-11	0.82	53.17	1.08E-08	0.44
539	100.92	2.28E-11	0.80	45.97	1.19E-08	0.45
544	84.81	2.12E-11	0.81	42.21	1.12E-08	0.43
550	73.23	2.25E-11	0.81	35.90	1.44E-08	0.43
554	64.75	2.31E-11	0.82	32.35	1.66E-08	0.42
559	59.66	2.53E-11	0.81	32.97	1.49E-08	0.44
563	48.90	2.58E-11	0.81	26.37	1.87E-08	0.42
568	42.97	2.43E-11	0.82	23.48	2.16E-08	0.41
574	37.11	2.50E-11	0.82	20.90	2.43E-08	0.40
579	32.78	2.69E-11	0.81	18.12	2.77E-08	0.40
583	28.35	2.61E-11	0.82	16.48	3.00E-08	0.39
588	25.62	3.07E-11	0.80	14.47	3.65E-08	0.39
593	25.62	3.07E-11	0.80	14.47	3.65E-08	0.39
598	19.82	2.97E-11	0.81	11.64	4.36E-08	0.38
603	17.06	2.57E-11	0.83	10.20	3.85E-08	0.38
607	15.56	3.04E-11	0.81	9.24	5.58E-08	0.37
612	13.72	3.01E-11	0.81	8.16	6.19E-08	0.36
618	12.07	2.99E-11	0.82	7.30	7.10E-08	0.36
622	10.84	3.08E-11	0.82	6.49	8.07E-08	0.35
627	9.63	3.07E-11	0.82	5.71	9.02E-08	0.35
633	8.49	3.23E-11	0.81	4.91	1.07E-07	0.35
637	7.67	3.24E-11	0.82	4.42	1.21E-07	0.35
642	6.88	3.20E-11	0.82	3.99	1.33E-07	0.34
648	6.07	3.28E-11	0.82	3.46	1.64E-07	0.34
652	5.50	3.32E-11	0.82	3.13	1.79E-07	0.34
657	4.97	3.36E-11	0.82	2.80	2.05E-07	0.33
662	4.45	3.41E-11	0.82	2.50	2.34E-07	0.33
667	3.94	3.50E-11	0.82	2.19	2.73E-07	0.32
673	3.52	3.48E-11	0.82	1.94	3.08E-07	0.32
677	3.22	3.56E-11	0.82	1.76	3.51E-07	0.32
682	2.90	3.61E-11	0.82	1.58	3.98E-07	0.31
687	2.61	3.73E-11	0.82	1.41	4.49E-07	0.31
692	2.34	3.79E-11	0.82	1.26	5.09E-07	0.31
697	2.10	3.87E-11	0.82	1.12	5.84E-07	0.31
702	1.89	3.94E-11	0.82	1.00	6.58E-07	0.30
707	1.69	3.98E-11	0.82	0.89	7.48E-07	0.30
712	1.52	4.01E-11	0.82	0.80	8.42E-07	0.29
717	1.36	4.06E-11	0.82	0.73	9.57E-07	0.29
736	0.86	4.49E-11	0.82	0.43	1.45E-06	0.28
742	0.76	4.47E-11	0.82	0.38	1.52E-06	0.28
746	0.69	4.49E-11	0.82	0.35	1.69E-06	0.28
751	0.62	4.52E-11	0.82	0.33	1.84E-06	0.27
756	0.56	4.58E-11	0.82	0.30	1.97E-06	0.27
760	0.51	4.53E-11	0.83	0.28	2.10E-06	0.27
765	0.47	4.53E-11	0.83	0.26	2.22E-06	0.26
